# Effects of sacubitril‐valsartan in the treatment of chronic heart failure patients with end‐stage renal disease undergoing dialysis

**DOI:** 10.1002/clc.24075

**Published:** 2023-06-28

**Authors:** Xiaoyan Liu, Lidong Huang, Gary Tse, Tong Liu, Jingjin Che

**Affiliations:** ^1^ Tianjin Key Laboratory of Ionic‐Molecular Function of Cardiovascular Disease, Department of Cardiology, Tianjin Institute of Cardiology the Second Hospital of Tianjin Medical University Tianjin China

**Keywords:** chronic heart failure, dialysis, end‐stage renal disease, sacubitril‐valsartan

## Abstract

**Background:**

The data on the effects of the angiotensin receptor–neprilysin inhibitor (ARNI) sacubitril‐valsartan (LCZ696) in chronic heart failure (CHF) patients with end‐stage renal disease (ESRD) requiring dialysis are lacking. This study assessed the efficacy and safety of LCZ696 in CHF patients with ESRD on dialysis.

**Hypothesis:**

LCZ696 treatment can reduce rehospitalization rate for HF, delay the occurrence of rehospitalization for HF, and prolong the survival time.

**Methods:**

We retrospectively analyzed the clinical data of CHF patients with ESRD on dialysis who were admitted to the Second Hospital of Tianjin Medical University from August 2019 to October 2021.

**Results:**

Sixty‐five patients had primary outcome during the follow‐up. The incidence of rehospitalization for HF in the control group was significantly higher than that in the LCZ696 group (73.47% vs. 43.28%, *p* = .001). There was no significant difference in mortality between the two groups (8.96% vs. 10.20%, *p* = 1.000). Our study included a time‐to‐event analysis through 1 year for the primary outcome—Kaplan–Meier curve showed that the LCZ696 group had significantly longer free‐event survival time than the control group over 1‐year follow‐up (median survival time 139.0 days vs. 116.0 days, *p* = .037).

**Conclusions:**

Our study found that LCZ696 treatment was associated with a reduction in HF rehospitalization without significant effects on serum creatinine and serum potassium levels. LCZ696 is effective and safe in CHF patients with ESRD on dialysis.

## Graphical abstract

Survival analysis curve (Kaplan–Meier). Kaplan–Meier curve showed that the LCZ696 group had significantly longer free‐event survival time than the control group over 1‐year follow‐up (median survival time 139.0 days vs. 116.0 days, *p* = .037).

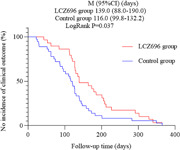

## 1 INTRODUCTION

According to left ventricular ejection fraction (LVEF), chronic heart failure (CHF) can be divided into HF with reduced ejection fraction (HFrEF), HF with preserved ejection fraction (HFpEF), and HF with mildly reduced ejection fraction (HFmrEF). Chronic kidney disease (CKD) has a high incidence in the general population, which is characterized by a gradual decrease in glomerular filtration rate (eGFR).^1^, ^2^ CKD often coexists with CHF, which may be due to their common and interacting risk factors, such as hypertension, diabetes, and hyperlipidemia, which increase the risks of adverse events.^3^, ^4^ End‐stage renal disease (ESRD), for which hemodialysis (HD) or peritoneal dialysis (PD) is required, accounts for a significant proportion of the CKD population.

As a first‐line drug for the treatment of CHF patients, angiotensin receptor–neprilysin inhibitor (ARNI) can increase the concentration of natriuretic peptide (ANP) by inhibiting enkephalinase (NEP), which can play a greater diuretic effect than angiotensin‐converting enzyme inhibitor (ACEI)/angiotensin receptor blocker (ARB). Previous studies have reported the cardiovascular benefits of ARNIs in heart failure and cancer patients.^5^, ^6^, ^7^ However, it is not known whether ARNIs provide similar benefits for ESRD patients who produce almost no urine. Previous studies have shown that the effects of ARNI on CKD patients are mainly reflected in four aspects: urinary protein, eGFR, blood pressure (BP), and cardiac function, although there are different opinions on the effect of urinary protein, it has an obvious protective effect on eGFR, BP, and cardiac function.^8^ Of these, the benefits on eGFR, BP, and cardiac function are more clear cut. A retrospective study of HFrEF patients with ESRD on dialysis has confirmed that LCZ696 can improve the levels of LVEF and cardiovascular biomarkers.^9^ A secondary analysis of renal function in PARADIGM‐HF showed that compared with RAAS inhibition alone, LCZ696 had a beneficial effect on the cardio‐renal system in patients with HFrEF, slowing down the decline of eGFR although accompanied by a slight increase in urinary protein.^10^ A real‐world study from Taiwan has shown that LCZ696 can reduce the risk of major cardiovascular events by 28% in CKD stage 4–5 patients with HFrEF.^11^ In the 2019 KDIGO Conference consensus paper, ARNI was listed as a cornerstone drug for the treatment of HFrEF with CKD and is considered to be available for dialysis patients with HFrEF.^12^ Regarding HFpEF, A retrospective study has shown that the administration of LCZ696 in ESRD patients with HFpEF can improve the level of cardiovascular biomarkers and cardiac function, which provides direct evidence for the safety and efficacy of ARNI in this kind of population.^13^ The 2022 Chinese Guidelines for the management of CHF in dialysis patients have included ARNI in the treatment of CHF with ESRD, but the quality of evidence remains in class C recommendations, and there is a lack of treatment data for the HFmrEF population.^14^ Given the paucity of data on renal patients, this study assessed the efficacy and safety of LCZ696 in CHF patients with ESRD on dialysis.

## 2 METHODS

### 2.1 Study design and patients

This study is a retrospective cohort study of CHF patients with ESRD on dialysis who were admitted to the Department of Cardiology, Department of Nephrology, and Department of Blood Purification in the Second Hospital of Tianjin Medical University from August 2019 to October 2021. The inclusion criteria were as follows: (1) Patients aged ≥ 18 years were hospitalized for an episode of acute decompensated heart failure (ADHF; due to deterioration of CHF, NYHA II–IV) and well on discharge; (2) received in‐hospital LCZ696 for the first time, (the LCZ696 group) or did not receive any RAAS inhibitors (the control group); and (3) the patients were complicated with ESRD, receiving HD or PD.

The exclusion criteria were: (1) in‐hospital death; (2) severe mitral or aortic stenosis; (3) infective endocarditis and acute myocarditis. (4) constrictive pericarditis or massive pericardial effusion; (5) malignant tumor or connective tissue disease; and (6) women during pregnancy.

The patients were admitted for an episode of ADHF and discharged following the completion of treatment. They were divided into the LCZ696 group and the control group (without any RAAS blocker) according to whether or not they were treated with LCZ696 after admission.

### 2.2 Data collection

We defined clinical outcome as rehospitalization for HF or all‐cause death, and recorded whether the patients had clinical outcome within 1 year. Biomarkers of cardiac and renal function were also collected: N‐Terminal Pro‐Brain Natriuretic Peptide (NT‐proBNP), serum creatinine (SCr), and Serum potassium. The ECG parameter (Rv_5_ + Sv_1_) was used as an indicator of the degree of left ventricular hypertrophy (LVH) degree.^15^

## 3 RESULTS

### 3.1 Study cohort and their baseline characteristics

From August 2019 to October 2021, a total of 1312 CHF patients with CKD were hospitalized at our hospital. Amongst these, 182 CHF patients with ESRD on dialysis were selected according to the inclusion and exclusion criteria. Finally, a total of 116 patients (excluding patients with incomplete date—21 cases and changed medication regimen—45 cases) completed retrospective follow‐up (LCZ696 group: *n* = 67, control group: *n* = 49).

Their baseline demographic and clinical characteristics are shown in Table 1. There were significant differences in systolic blood pressure (LCZ696 group vs. control group: 157.00 ± 22.76 mmHg vs. 145.47 ± 20.71 mmHg, *p* = .006) and diastolic blood pressure (LCZ696 group vs. control group: 85.75 ± 12.72 mmHg vs. 80.06 ± 11.51 mmHg, *p* = .015) between the two groups. The BP in the LCZ696 group was significantly higher than that in the control group. The baseline level of NT‐proBNP in the LCZ696 group was higher than that in the control group (*p* = .030). The LCZ696 group has more coronary heart disease patients than the control group (82.1% vs. 63.3%, *p* = .022).

**Table 1:** Baseline characteristics of the two groups.

Variables	LCZ696 group (*n* = 67)	Control group (*n* = 49)	*p* Value
Gender			
Female	32 (47.8%)	22 (44.9%)	.760
Male	35 (52.2%)	27 (55.1%)	
Age (years)	59.25 ± 12.45	62.98 ± 13.31	.125
Duration of dialysis (months)	5.00 (47.00)	2.00 (49.00)	.923
Dialysis method			
PD	42 (62.7%)	35 (71.4%)	.325
HD	25 (37.3%)	14 (28.6%)	
SBP (mmHg)	157.00 ± 22.76	145.47 ± 20.71	.006
DBP (mmHg)	85.75 ± 12.72	80.06 ± 11.51	.015
HR (bpm)	79.30 ± 13.89	80.92 ± 11.94	.512
NT‐proBNP (pg/mL)	13643.10 (12298.60)	10207.70 (24538.60)	.030
SCr (μmol/L)	692.70 (444.50)	735.30 (516.20)	.909
Serum potassium (mmol/L)	4.51 ± 0.75	4.41 ± 0.84	.494
CHD	55 (82.1%)	31 (63.3%)	.022
Cerebral infarction	12 (17.9%)	10 (20.4%)	.735
Diabetes	32 (47.8%)	19 (38.8%)	.335
Hypertension	67 (100.0%)	46 (93.9%)	.144
AF	3 (6.0%)	4 (8.2%)	.929
NYHA class II	32 (47.8%)	29 (59.2%)	
III	24 (35.8%)	16 (32.7%)	.320
IV	11 (16.4%)	4 (8.2%)	
Antidiabetic drugs	6 (9.0%)	5 (10.2%)	1.000
CCB	53 (79.1%)	31 (63.3%)	.059
β blockers	37 (55.2%)	21 (42.9%)	.188
MRA	3 (4.5%)	2 (4.1%)	1.000
Diuretic	3 (4.5%)	4 (8.2%)	.668
Statins	12 (18.2%)	10 (20.4%)	.764
Digitalis	1 (1.5%)	10 (20.4%)	1.000
Antiplatelet drugs	20 (29.9%)	10 (20.4%)	.251

*Note*: Data are mean (SD), number (%), or median (IQR).

Abbreviations: AF, atrial fibrillation; CCB, calcium channel blocker; CHD, coronary heart disease; DBP, diastolic blood pressure; HD, hemodialysis; HR, hazard ratio; IQR, interquartile range; LCZ696, sacubitril‐valsartan; MRA, aldosterone antagonist; NT‐proBNP, N‐terminal pro‐BNP; NYHA, New York Heart Association; PD, peritoneal dialysis; SBP, systolic blood pressure; SCr, serum creatinine.

### 3.2 Effect of LCZ696 treatment on primary outcome

**Figure 1:**
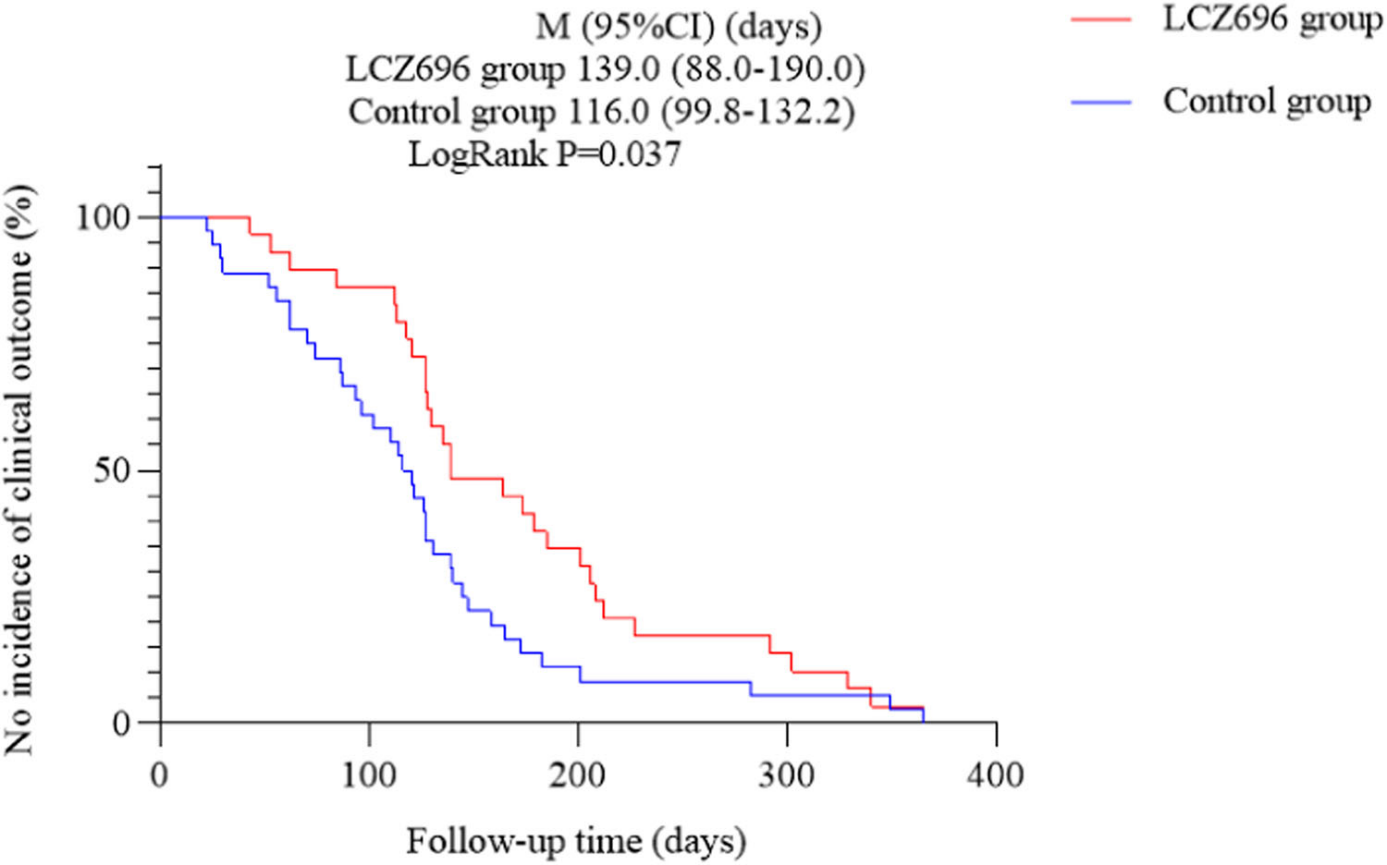
Survival analysis curve (Kaplan–Meier). CI, confidence interval; LCZ696, sacubitril‐valsartan.

**Figure 2:**
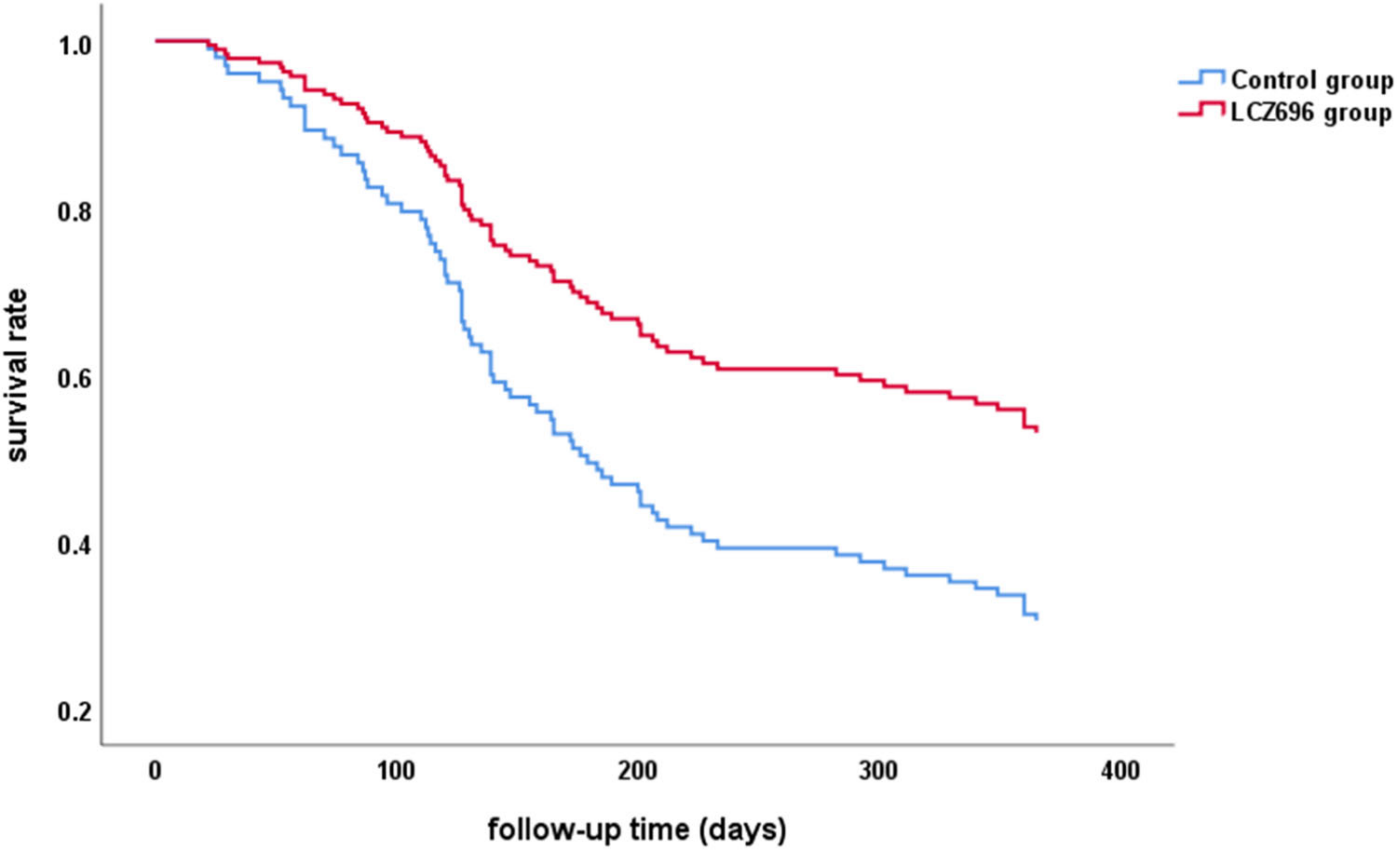
Survival analysis curve (Cox regression). LCZ696, sacubitril‐valsartan.

During follow‐up, 65 patients in total met the primary outcome of either rehospitalization for HF or all‐cause death (LCZ696 group: *n* = 29; control group: *n* = 36). The incidence of rehospitalization for HF in the control group was significantly higher than that in the LCZ696 group (73.47% vs. 43.28%, *p* = .001) with no significant difference in the mortality (8.96% vs. 10.20%, *p* = 1.000) (Table 2). This study included a time‐to‐event analysis^16^ through 1 year for the primary outcome—Kaplan–Meier analysis showed that the free‐event survival time of the LCZ696 group (median survival time 139.0 days, 95% CI = 88.0–190.0) was significantly longer than that of the control group (median survival time 116.0 days, 95% CI = 99.8–132.2) during the 1‐year follow‐up (log‐rank test: *p* = .037; Figure 1). We analyzed 21 patients with lack of laboratory data (9 patients in the LCZ696 group and 12 patients in the control group) and 116 patients with complete data. COX regression survival curve showed that the survival condition of the LCZ696 group was better than that of the control group (Figure 2).

**Table 2:** The incidence of clinical outcome of the two groups.

Clinical outcome	LCZ696 group (*n* = 67)	Control group (*n* = 49)	*p* Value
Rehospitalization for HF	29 (43.28%)	36 (73.47%)	0.001
All‐cause death	6 (8.96%)	5 (10.20%)	1.000

*Note*: Data are mean (SD), number (%), or median (IQR).

Abbreviations: HF, heart failure; IQR, interquartile range; LCZ696, sacubitril‐valsartan.

### 3.3 Risk factors of the primary outcome

**Figure 3:**
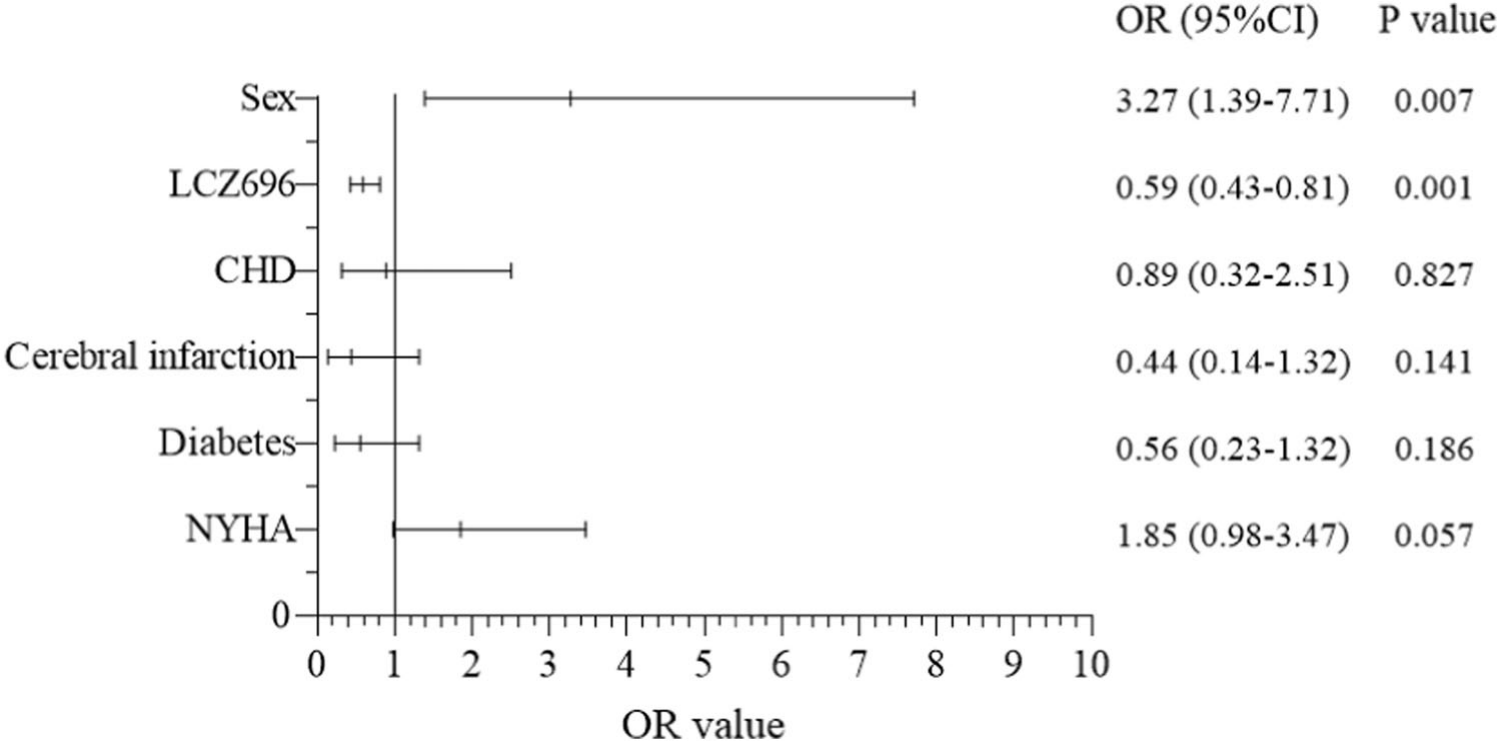
Logistic regression to identify significant predictors of the primary outcome. CHD, coronary heart disease; CI, confidence interval; LCZ696, sacubitril‐valsartan; NYHA, New York Heart Association; OR, odds ratio.

Significant differences in gender (*p* = .002, *p* < .05), cerebral infarction (*p* = .039) and LCZ696 (*p* = .001) were observed between the LCZ696 and control groups at baseline (Table 3). The significant variables were included in the multivariable logistic regression model, demonstrating that female gender (hazard ratio [HR] = 3.27, 95% CI = 1.39–7.71, *p* = .007) and LCZ696 were significant predictors of the primary outcome (HR = 0.59, 95% CI = 0.43–0.81, *p* = .001; Figure 3). Cox regression showed that LCZ696 treatment was an obstacle to primary outcome occurrence (HR = 0.81, 95% CI = 0.70‐0.95, *p* = .007; Table 4).

### 3.4 Effect of LCZ696 treatment on NT‐proBNP, R_v5_ + S_v1_, SCr, and serum potassium levels

No significant difference in the changes of NT‐proBNP, R_v5_ + S_v1_, SCr, and serum potassium levels was observed between the two groups during the 1‐year follow‐up (Table 5).

## 4 DISCUSSION

This was a retrospective study of LCZ696‐treated CHF patients with ESRD on dialysis. To the best of the authors' knowledge, there are to date two retrospective studies of LCZ696 treatment on patients with both HF and ESRD on dialysis, with very limited sample size of 23 HFrEF patients^9^ and 21 HFpEF patients.^13^ The reason may be that the number of such patients is small in clinical practice and the data is difficult to collect. The results of the two studies are complementary to each other, suggesting that ARNI can improve cardiovascular markers and cardiac function in patients with HFrEF and HFpEF complicated with dialysis, but neither of the two studies deals with the analysis of the effects of ARNI on the survival status of patients. Our study included a range of CHF patients across different LVEFs who were also on dialysis for ESRD. The results showed that LCZ696 treatment can reduce rehospitalization rate for HF, delay the occurrence of rehospitalization for HF, and prolong the survival time. LCZ696 was the protective factor for the occurrence of rehospitalization for HF or all‐cause death. LCZ696 was relatively safe in terms of renal function and electrolytes.

The pathophysiology of HF in CKD progressing to ESRD is very complex and includes multiple mechanisms from renal impairment: uncontrolled hypertension, LVH and fibrosis, excessive preload and afterload, neurohormonal activation, and anemia.^13^ Currently, neurohormones are considered to play a key role in the progression of HF in CKD patients. ARNI plays a role by enhancing the natriuretic peptide system and neuroendocrine mechanism (inhibiting RAAS and sympathetic nervous system activity), which can play a better diuretic effect than ACEI/ARB to reduce load and antagonize neurohormone to reverse ventricular remodeling.^8^ Therefore, increasing attention has been paid to the role of ARNI in HF patients complicated with CKD. For ESRD in dialysis patients, a study had shown that LCZ696 treatment can increase short‐term peritoneal ultrafiltration in PD patients, but did not increase 24 h urine volume, which demonstrated the role of LCZ696 in water removal and BP control in PD patients.^17^ Therefore, it is possible for LCZ696 to play a beneficial role in CHF patients with ESRD on dialysis in clinical treatment.

Regarding cardiovascular outcomes in HF patients with CKD, in the PARADIGM‐HF trial, LCZ696 reduced the risk of major composite endpoints (cardiovascular disease‐related deaths and HF hospitalization) by 20% and all‐cause mortality by 16% compared with enalapril, and eGFR (30–60 mL/min/1.73 m^2^) and urinary albumin/creatinine ratio did not affect the improvement of LCZ696 on HF hospitalization and cardiovascular‐related deaths.^18^, ^19^, ^20^ However, large clinical prospective trials such as PARADIGM‐HF all exclude ESRD on dialysis patients, and thus the effects of LCZ696 on HF hospitalization and mortality in CHF patients with ESRD remain uncertain. In our study, 65 patients met the primary outcome of rehospitalization for HF or all‐cause death during the follow‐up. Logistic regression analysis showed that LCZ696 was the protective factor for the occurrence of clinical outcome. The incidence of rehospitalization for HF in the LCZ696 group was significantly lower than that in the control group (*p* = .001). Survival analysis showed that LCZ696 treatment can reduce rehospitalization rate for HF, delay the occurrence of rehospitalization for HF, and prolong the survival time (median survival time 139.0 days vs. 116.0 days). Although there were few clinical outcome events observed and the morality had not reached a statistical difference between the two groups because of only 1‐year follow‐up, this result also confirmed the effectiveness of LCZ696 treatment in CHF patients with ESRD on dialysis in real‐world settings.

In a meta‐analysis of 27 long‐term prospective studies in ESRD patients, the increase of BNP/NT‐proBNP was significantly associated with increased all‐cause mortality, cardiovascular mortality, and cardiovascular events.^21^ NT‐proBNP is less affected by ARNI than BNP, so this study chose NT‐proBNP as the laboratory index to evaluate cardiac function, but the only way for NT‐proBNP clearance is glomerular filtration, and renal insufficiency has a great impact on it, so it is necessary to dynamically evaluate NT‐proBNP level changes.^22^, ^23^ With the decline of renal function, cardiac structural remodeling will also occur, mainly characterized by LVH, which increases with the decline of eGFR, up to 90% in ESRD patients.^24^ The ECG parameter (Rv_5_ + Sv_1_) was included in the study to represent the degree of LVH. The results showed that there was no significant improvement in NT‐proBNP level and Rv_5_ + Sv_1_ amplitude in the LCZ696 group compared with the control group during the 1‐year follow‐up visit. For the evaluation of cardiac structure and function, ultrasonic cardiogram (UCG) will be more convincing than ECG parameters. However, we could not analyze UCG data due to the limited data in this study, which shall be the focus of our next research study. The above indicators may get meaningful results with the extension of follow‐up time, the further improvement of data, and the dynamic evaluation of multiple observation points.

In clinical practice, potential deterioration of renal function is often the biggest limiting factor for prescribing LCZ696 in CKD patients. Thus, the safety of LCZ696 in renal function and electrolytes has also attracted much attention in various studies. In the subgroup analysis of PARADIGM‐HF and PARAMOUNT‐HF trails, it was found that LCZ696 delayed the deterioration of renal function in patients with HFrEF or HFpEF compared with RAAS inhibitors, but eGFR persisted in 30–60 mL/min/1.73 m^2^.^25^, ^26^ The UK HARP‐III trial demonstrated that in CKD (GFR 20–60 mL/min/1.73 m^2^) patients that LCZ696 had no additional protective effect on kidney function or albuminuria compared with irbesartan, but it lowered BP and cardiac biomarker levels.^27^ Therefore, the effects of LCZ696 on renal function in overall CKD patients require further study, especially in ESRD patients. Our results showed that LCZ696 treatment did not have a significant effect on SCr and serum potassium levels in CHF patients with ESRD on dialysis. This may be because ESRD patients only have residual renal function or total loss of renal function, and therefore SCr and serum potassium levels did not show significant improvement during follow‐up. Nevertheless, the use of LCZ696 in ESRD patients was relatively safe in terms of renal function and electrolytes.

Several limitations of this study should be recognized. First, the study is a single‐center retrospective study with a small sample size and short follow‐up time. It was therefore difficult to assess the long‐term changes of cardiac and renal function. Second, this study is a retrospective cohort study with unmeasured confounding factors (including duration of HF, HF types, and etiology of HF and CKD). Finally, all the patients selected in this study are ESRD on dialysis patients, and thus the effects of volume load could not be excluded. Therefore, larger prospective, long‐term follow‐up studies including RCT should be warranted. At the same time, prospective and retrospective studies in the real world are also needed to provide clinical evidence in the future.

## 5 CONCLUSIONS

Our study found that LCZ696 treatment was associated with a reduction in HF rehospitalization without significant effects on mortality, NT‐proBNP, and R_v5_ + S_v1._ LCZ696 is safe in CHF patients with ESRD on dialysis in terms of SCr and serum potassium levels.

## AUTHOR CONTRIBUTIONS

*Collect data and wrote the paper*: Xiaoyan Liu and Lidong Huang. *Detailed instruction, edited and reviewed the manuscript*: Gray Tse, Jingjin Che, and Tong Liu. *Final approval of manuscript*: all authors.

## CONFLICT OF INTEREST STATEMENT

The authors declare no conflict of interest.

## Supporting information

Supporting information.

## Data Availability

The data that support the findings of this study are available from the corresponding author upon reasonable request.
