# Supplementary material for: Effects of sacubitril‐valsartan in the treatment of chronic heart failure patients with end‐stage renal disease undergoing dialysis
Source: Clin Cardiol. 2023 Jun 28;46(8):930–6. doi: 10.1002/clc.24075 (PMC10436793; doi:10.1002/clc.24075)
Supplement: Supplementary file 1 — Supporting information. [file CLC-46-930-s001.docx]

Table 3 Comparisons of baseline characteristics between patients who developed the primary outcome and those who did not

| Variables | Event group (n=65) | Free-event group (n=51) | *P-*value |
| --- | --- | --- | --- |
| Gender male  female | 43 (66.2%)  22 (33.8%) | 19 (37.3%)  32 (62.7%) | 0.002 |
| Age (years)  < 65  ≥ 65 | 39 (60.0%)  26 (40.0%) | 26 (51.0%)  25 (49.0%) | 0.331 |
| Duration of  Dialysis (months) | 3.00 (24.00) | 5.00 (60.00) | 0.722 |
| Dialysis method  PD  HD | 42 (64.6%)  23 (35.4%) | 35 (68.6%)  16 (31.4%) | 0.650 |
| CHD | 45 (69.2%) | 41 (80.4%) | 0.173 |
| Cerebral infarction | 8 (12.3%) | 14 (27.5%) | 0.039 |
| Diabetes | 25 (38.5%) | 26 (51.0%) | 0.178 |
| Hypertension | 62 (95.4%) | 51 (100.0%) | 0.334 |
| AF | 6 (9.2%) | 2 (3.9%) | 0.453 |
| NYHA class II  III  IV | 30 (46.2%)  24 (36.9%)  11 (16.9%) | 31 (60.8%)  16 (31.4%)  4 (7.8%) | 0.198 |
| LCZ696 | 29 (44.6%) | 38 (74.5%) | 0.001 |

Data are mean (SD), number (%), or median (IQR). PD=Peritoneal dialysis. HD=Hemodialysis. CHD=Coronary heart disease. AF=[Atrial fibrillation](javascript:;). NYHA=New York Heart Association. LCZ696=Sacubitril-Valsartan.

Table 4 Multivariate COX regression analysis (N=137)

| Variables | HR | 95% CI | *P-*value |
| --- | --- | --- | --- |
| Gender female | 0.83 | 0.53-1.31 | 0.420 |
| CHD | 0.99 | 0.60-1.63 | 0.955 |
| Cerebral infarction | 1.25 | 0.73-2.17 | 0.420 |
| Diabetes | 1.04 | 0.66-1.64 | 0.860 |
| NYHA class | 1.08 | 0.79-1.48 | 0.619 |
| LCZ696 | 0.81 | 0.70-0.95 | 0.007 |

CHD=Coronary heart disease. NYHA=New York Heart Association. LCZ696=Sacubitril-Valsartan.

Table 5 Biomarkers and Rv_5_+Sv_1_ amplitude of the two groups

|  | LCZ696 group (n=67) | Control group (n=49) | Median difference  (95% CI) | *P* |
| --- | --- | --- | --- | --- |
| NT-proBNP  (pg /mL) |  |  |  |  |
| Baseline | 13643.10  (12298.60) | 10207.70  (24538.60) |  |  |
| After 1 year | 15614.30  (26555.00) | 10916.10  (29847.00) |  |  |
| Difference | 0.00（7395.75） | -368.6（12703.15） | 0.00 (-2871.70-2473.30) | 0.773 |
| SCr (μmol /L) |  |  |  |  |
| Baseline | 692.70 (444.50) | 735.30 (516.20) |  |  |
| After 1 year | 790.76 ± 320.99 | 883.37 ± 364.46 |  |  |
| Difference | -3.70（344.03） | 54.70（361.11） | 21.50 (-31.10-158.90) | 0.157 |
| Serum potassium (mmol /L) |  |  |  |  |
| Baseline | 4.51 ± 0.75 | 4.41 ± 0.84 |  |  |
| After 1 year | 4.40 (1.20) | 4.00 (1.60) |  |  |
| Difference | -0.13±0.96 | -0.16±1.22 | -5.91 (-19.55-7.73) | 0.393 |
| Rv_5_+Sv_1_ (uV) |  |  |  |  |
| Baseline | 2250.88 ± 1057.62 | 2255.92 ± 914.22 |  |  |
| After 1 year | 2198.36 ± 973.64 | 2123.08 ± 977.20 |  |  |
| Difference | -52.52±901.99 | -133.56±946.45 | -81.0 (-425.99-263.91) | 0.643 |

Data are mean (SD), number (%), or median (IQR). NT-proBNP =N-terminal pro-BNP. SCr=serum creatinine.
